# Body temperature affects cerebral hemodynamics in acutely brain injured patients: an observational transcranial color-coded duplex sonography study

**DOI:** 10.1186/s13054-014-0552-7

**Published:** 2014-10-14

**Authors:** Federica Stretti, Miriam Gotti, Silvia Pifferi, Giovanna Brandi, Federico Annoni, Nino Stocchetti

**Affiliations:** Neuroscience ICU, Fondazione IRCCS Ca’ Granda Ospedale Maggiore Policlinico, Milan, Italy; Department of Pathophysiology and Transplantation, Università degli Studi di Milano, Milan, Italy; Chirurgische Intensivstation, UniversitätsSpital Zürich, Zürich, Switzerland; Department of Surgery, Fondazione IRCCS Ca’ Granda Ospedale Maggiore Policlinico, Milan, Italy

## Abstract

**Introduction:**

Temperature changes are common in patients in a neurosurgical intensive care unit (NICU): fever is frequent among severe cases and hypothermia is used after cardiac arrest and is currently being tested in clinical trials to lower intracranial pressure (ICP). This study investigated cerebral hemodynamics when body temperature varies in acute brain injured patients.

**Methods:**

We enrolled 26 patients, 14 with acute brain injury who developed fever and were given antipyretic therapy (defervescence group) and 12 who underwent an intracranial neurosurgical procedure and developed hypothermia in the operating room; once admitted to the NICU, still under anesthesia, they were re-warmed before waking (re-warming group). We measured cerebral blood flow velocity (CBF-V) and pulsatility index (PI) at the middle cerebral artery using transcranial color-coded duplex sonography (TCCDS).

**Results:**

In the defervescence group mean CBF-V decreased from 75 ± 26 (95% CI 65 to 85) to 70 ± 22 cm/s (95% CI 61 to 79) (*P* = 0.04); the PI also fell, from 1.36 ± 0.33 (95% CI 1.23 to 1.50) to 1.16 ± 0.26 (95% CI 1.05 to 1.26) (*P* = 0.0005). In the subset of patients with ICP monitoring, ICP dropped from 16 ± 8 to 12 ± 6 mmHg (*P* = 0.003). In the re-warming group mean CBF-V increased from 36 ± 10 (95% CI 31 to 41) to 39 ± 13 (95% CI 33 to 45) cm/s (*P* = 0.04); the PI rose from 0.98 ± 0.14 (95% CI 0.91 to 1.04) to 1.09 ± 0.22 (95% CI 0.98 to 1.19) (*P* = 0.02).

**Conclusions:**

Body temperature affects cerebral hemodynamics as evaluated by TCCDS; when temperature rises, CBF-V increases in parallel, and viceversa when temperature decreases. When cerebral compliance is reduced and compensation mechanisms are exhausted, even modest temperature changes can greatly affect ICP.

## Introduction

Temperature changes are common in patients in a neurosurgical intensive care unit (NICU) where fever is frequent, especially in the most severe patients [1]. In fact, hypothermia is applied for survivors of cardiac arrest and is currently being tested in clinical trials to lower intracranial pressure (ICP) after traumatic brain injury [2].

In experimental studies fever has negative effects on the brain, because it increases metabolism, glutamate release, free radical production and blood-brain barrier permeability, and worsening edema [3]. Of additional concern is the effect on ICP. Our group [4] simultaneously measured brain temperature (T_brain_) and ICP, showing that ICP rises with fever onset and declines after defervescence; others subsequently confirmed this relationship [5,6]. Conversely, when hypothermia is applied the cerebral metabolism is slowed, and cerebral blood flow (CBF) falls. These effects both contribute to lower ICP [7]. The relationships between temperature and CBF are therefore of clinical interest, in view of the parallel changes in ICP.

Ideally, CBF at various T_brain_ should be explored but unfortunately there are obvious limitations to this: T_brain_ requires special probes, and some techniques marketed for CBF monitoring induce T_brain_ changes themselves in order to quantify local CBF, so they do not seem appropriate for assessing CBF in response to spontaneous changes. A different technique, transcranial color-coded duplex sonography (TCCDS), measures CBF velocity (CBF-V) in a particular tract of a specific vessel. From this measurement, CBF is estimated according to a mathematical equation [8].

Core body temperature (T_core_) is the most widely available proxy of brain temperature itself [4]. In this study we investigated cerebral hemodynamics using TCCDS, at different body temperatures in patients with acute brain injury. We explored two conditions: (1) defervescence, when febrile patients were treated with antipyretic drugs and (2) re-warming, after intracranial neurosurgical procedures.

## Materials and methods

The study involved 26 patients admitted to the NICU at the Ospedale Maggiore Policlinico of Milan. Of these patients, 14 (8 male, 6 female, age 54 ± 16 years) with acute brain injury developed fever and were given antipyretic therapy (defervescence group), and 12 patients (7 male, 5 female, age 56 ± 14) underwent an intracranial neurosurgical procedure under general anesthesia and suffered unexpected hypothermia; these patients were re-warmed once admitted to the NICU, before waking (re-warming group). Their main epidemiological data are shown in Tables 1 and 2.

**Table 1:** **Main epidemiological data: defervescence group**

**Patient**	**Pathology**	**ICP catheter**	**GCS m**	**Starting T**_**core**_ **(°C)**	**Final T**_**core**_ **(°C)**	**Antipyretic drug**	**Physical therapy**
1	TBI	+	1	38.9	38.3	+	+
2	ICH	+	6	38.5	37.5	+	+
3	SAH	+	6	38.5	37.2	+	+
4	TBI	+	2	39.1	37.9	+	-
5	SAH	-	6	39.0	37.7	+	-
6	TBI	+	3	38.8	38.0	+	-
7	TBI	-	6	39.3	38.2	+	-
8	TBI	-	5	39.9	38.4	+	-
9	TBI	-	6	38.8	37.3	+	-
10	SAH	-	6	39.5	38.5	+	+
11	TBI	+	5	39.1	37.9	+	-
12	SAH	+	5	38.9	37.9	+	-
13	SAH	+	6	39.3	38.2	+	-
14	ICH	-	5	39.8	38.8	+	-

TBI, traumatic brain injury; ICH, intraparenchymal hemorrhage; SAH subarachnoid hemorrhage, ICP, intracranial pressure; GCSm Glasgow coma scale motor response; T_core_ core temperature.

**Table 2:** **Main epidemiological data: re-warming group**

**Patient**	**Pathology**	**Starting T**_**core**_ **(°C)**	**Final T**_**core**_ **(°C)**
1	Intra-axial neoplasm	33.7	34.7
2	Intra-axial neoplasm	35.5	36.2
3	Intra-axial neoplasm + aneurysm	35.5	36.5
4	Intra-axial neoplasm	35.9	36.6
5	Extra-axial neoplasm	36.5	37.2
6	Intra-axial neoplasm	35.5	36.5
7	Intra-axial neoplasm	36.3	37.0
8	Aneurysm	36.3	37.1
9	Extra-axial neoplasm	35.4	36.3
10	Aneurysm	36.1	36.7
11	Intra-axial neoplasm	35.0	35.7
12	Extra-axial neoplasm	34.5	35.5

T_core_ core temperature.

T_core_ was measured in the bladder or the pharynx. None of the other parameters, such as the ventilatory setting and drug infusions (particularly sedatives and analgesics), were modified during the study.

### Defervescence group

Data were collected at two times: with fever (defined as a T_core_ ≥38.4°C [9]) and after defervescence (a temperature reduction of at least 0.7°C) induced by antipyretic drugs, acetaminophen or diclofenac (Table 1). Eight patients had ICP monitoring and we calculated the cerebral perfusion pressure (CPP) as:

$$ \mathrm{C}\mathrm{P}\mathrm{P} = \mathrm{MAP}\ \hbox{--}\ \mathrm{I}\mathrm{C}\mathrm{P} $$

where MAP is mean arterial pressure. In six patients the internal jugular bulb was cannulated for intermittent determination of oxygen saturation in the jugular vein (S_j_O_2_).

### Re-warming group

Patients in this group suffered an unexpected drop in T_core_ during general anesthesia. Data were collected immediately when they entered the NICU and when the temperature target, a T_core_ increase ≥0.7°C, was achieved by active warming. We used a convective air warming system connected to a full-body blanket (WarmTouch™ Convective Air Warming System, Covidien, Boulder, CO, USA). ICP and S_j_O_2_ were not monitored in this group. All patients had uneventful post-surgery recovery.

### TCCDS measurements

We used the iE33 xMATRIX Echography System (Koninklijke Philips Electronics NV, Amsterdam, The Netherlands) for TCCDS, measuring CBF-V and pulsatility index (PI) in the middle cerebral artery (MCA), at the M1 tract, the most proximal to the internal carotid artery bifurcation. The measurements were always taken at the same depth and insonation angle, with very accurate angle correction, in order to ensure they were comparable. We insonated the MCA bilaterally in 21 patients, and on the right side only in 3 patients, and on the left side in 2 patients, because there was no adequate controlateral insonation window. We insonated the MCA at a mean depth of 54 ± 3 mm on the right and 54 ± 7 mm on the left; the angle was 22 ± 19° on the right and 27 ± 13° on the left.

### Statistical analysis

We used two statistical software packages: Prism 5.00 (GraphPad Software, San Diego, CA USA) and SPSS 7.0 (IBM Software, Armonk, NY, USA). All data were normally distributed (checked with D’Agostino-Pearson omnibus normality test) so they are all summarized as mean ± standard deviation.

Our analysis consisted of two steps: (1) first, the paired *t*-test was used to compare TCCDS measurements before and after defervescence or warming; (2) then, we built a general linear model for repeated measures, in order to verify the effect of three independent variables on CBF-V changes: insonation side (right or left), method used to modify temperature (defervescence or re-warming) and temperature itself (T_core_). A probability (*P*-value) <0.05 was accepted as significant.

### Ethics

The interventions described (antipyretic treatment in the case of fever and re-warming after neurosurgery) are part of routine management. Monitoring, including continuous invasive ICP and intermittent TCCDS recording, is also part of the standard of care. No informed consent was sought for this specific study. Relatives of unconscious patients were informed in writing that monitoring data, once anonymized, might be used for research. Our hospital institutional ethics committee (*Comitato Etico of Fondazione IRCCS Ca’ Granda Ospedale Maggiore Policlinico*) was notified of the study, and approved it, waiving the need for consent.

## Results

### Defervescence group

**Figure 1:**
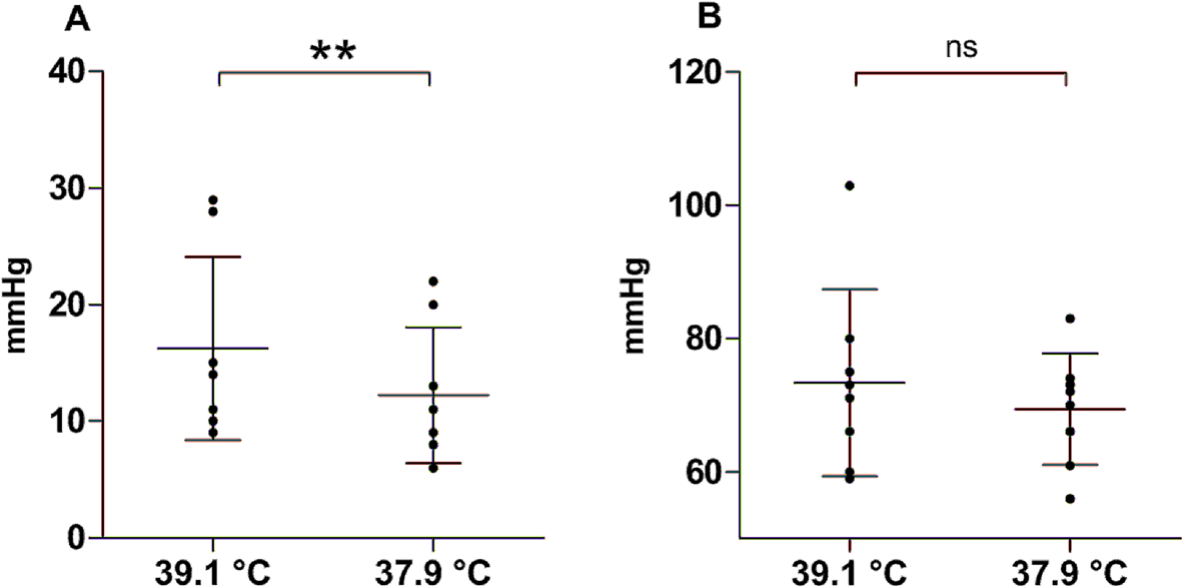
**Data are presented as mean ± standard deviation; every dot represents the value of the considered parameter in one patient. (A)** Mean difference in intracranial pressure (ICP) between the two time points: before (mean core temperature 39.1°C) and after defervescence (mean core temperature 37.9°C) (8 patients). **(B)** corresponding mean difference in cerebral perfusion pressure (CPP). Paired t-test: ns, not significant, **P <0.01.

**Figure 2:**
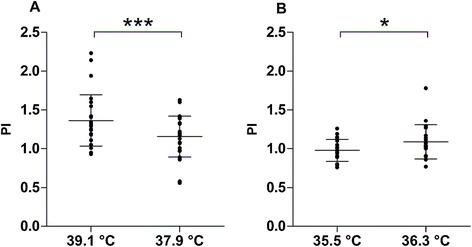
**Data are presented as mean ± standard deviation; every dot represents the value of the considered parameter in one artery. (A)** Mean difference in pulsatility index (PI) between the two time points in the defervescence group: during fever (mean core temperature 39.1°C) and after defervescence (mean core temperature 37.9°C) (14 patients). **(B)** Mean difference in PI between the two time points in the re-warming group: before (mean core temperature 35.5°C) and after re-warming (mean core temperature 36.3°C) (12 patients). Paired *t*-test. *P <0.05, ***P <0.001.

We studied patients on average 7 ± 6 days after brain injury. The mean T_core_ fell from 39.1 ± 0.4 to 37.9 ± 0.5°C. Cerebral monitoring showed a reduction in ICP from 16 ± 8 to 12 ± 6 mmHg (*P* = 0.003) (Figure 1A) although CPP did not change (Figure 1B). CBF-V decreased: mean velocity from 75 ± 26 (95% CI 65, 85) to 70 ± 22 cm/s (95% CI 61, 79) (*P* = 0.04) and systolic peak velocity from 144 ± 54 (95% CI 123, 166) to 126 ± 41 (95% CI 110, 142) cm/s (*P* = 0.002). The PI dropped from 1.36 ± 0.33 (95% CI 1.23, 1.50) to 1.16 ± 0.26 (95% CI 1.05, 1.26) (*P* = 0.0005) (Figure 2A).

Systemic monitoring showed a reduction in HR from 86 ± 17 to 80 ± 16 bpm (*P* = 0.006) and MAP from 91 ± 14 to 85 ± 15 mmHg (*P* = 0.02); other parameters were stable (Table 3).

**Table 3:** **Blood gases and systemic monitoring**

**Parameter**	**Defervescence group**			**Re-warming group**		
	**(n = 14)**			**(n = 12)**		
	***Before***	***After***	***P-value***	***Before***	***After***	***P-value***
**T**_**core**_ **(°C)**	39.1 ± 0.4	37.9 ± 0.5	<0.0001	35.5 ± 0.8	36.3 ± 0.7	<0.0001
**P**_**a**_ **CO**_**2**_ **(mmHg)**	33 ± 5	34 ± 5	0.08	33 ± 4	34 ± 5	0.43
**pH**_**a**_	7.48 ± 0.04	7.48 ± 0.04	0.15	7.45 ± 0.05	7.44 ± 0.07	0.80
**lactate (mmol/L)**	0.9 ± 0.3	0.9 ± 0.2	1.00	1.3 ± 0.8	1.1 ± 0.7	0.01
**MAP (mmHg)**	91 ± 14	85 ± 15	0.02	82 ± 9	91 ± 15	0.01
**HR (bpm)**	86 ± 17	80 ± 16	0.006	64 ± 12	67 ± 13	0.10

T_core_, core temperature; P_a_CO_2_, arterial carbon dioxide partial pressure; pH_a_, arterial pH; MAP, mean arterial pressure; HR, heart rate. The *P*-value is reported for each parameter.

### Re-warming group

On average, the T_core_ rose from 35.5 ± 0.8 to 36.3 ± 0.7°C. CBF-V increased: mean velocity from 36 ± 10 (95% CI 31, 41) to 39 ± 13 (95% CI 33, 45) cm/s (*P* = 0.04) and systolic peak velocity from 59 ± 18 (95% CI 51, 67) to 68 ± 20 (95% CI 58, 77) cm/s (*P* = 0.0004). The PI rose from 0.98 ± 0.14 (95% CI 0.91, 1.04) to 1.09 ± 0.22 (95% CI 0.98, 1.19) (*P* = 0.02) (Figure 2B).

Systemic monitoring showed an increase in MAP from 82 ± 9 to 91 ± 15 mmHg (*P* = 0.01) and a reduction in arterial lactate from 1.3 ± 0.8 to 1.1 ± 0.7 (*P* = 0.01); other parameters were stable (Table 3).

**Figure 3:**
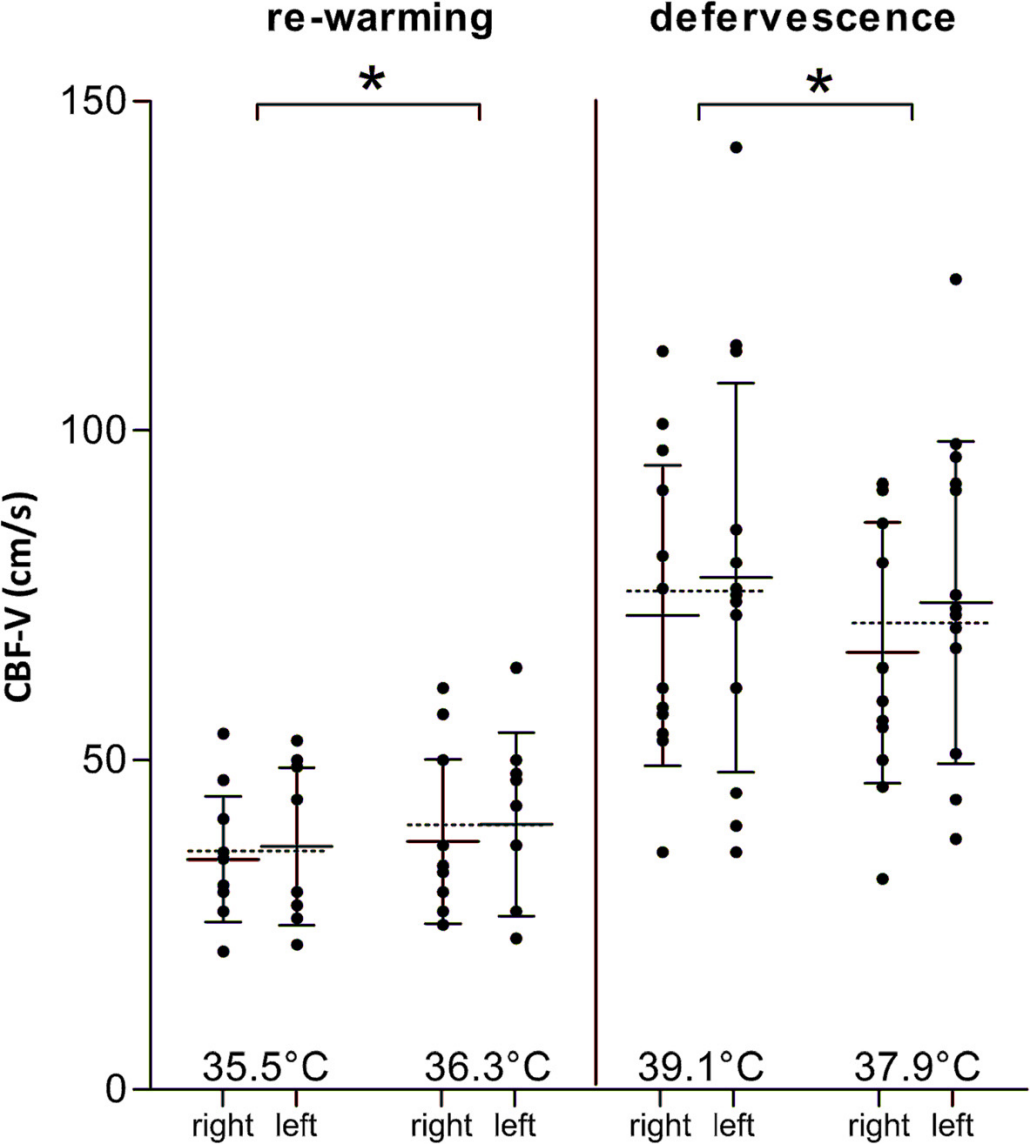
**General linear model for repeated measures.** Data are presented as mean ± standard deviation; every dot represents the value of the considered parameter in one artery. On the left, results for the re-warming group (12 patients); on the right, results for the defervescence group (14 patients). Mean cerebral blood flow velocity (CBF-V) is presented in separate columns, for insonation of the right and left middle cerebral artery. The dotted lines indicate the mean for each pair of columns. Temperature changes had an effect on CBF-V (*P* = 0.01). Insonation side and method used to modify temperature (defervescence or re-warming) did not significantly affect mean CBF-V changes.

The general linear model for repeated measures analysis confirmed that the only independent variable that explained CBF-V changes was T_core_ (*P* = 0.01); the other two independent variables (insonation side and method used to modify temperature) could not predict changes in CBF-V, by Greenhouse-Geisser, Huynh-Feldt or lower-bound analysis (Figure 3).

## Discussion

### Main findings

CBF-V decreased during defervescence in patients with acute brain damage, and rose during re-warming in a T_core_ range from 33.7 to 39.9°C. The PI also dropped during defervescence and rose during re-warming. CBF-V varied despite constant CPP, which remained stable because the MAP reduction was concurrent to the decrease in ICP during defervescence. SjO_2_, which reflects the balance between cerebral metabolic rate and delivery of oxygen, did not change during defervescence, suggesting that the relationship between metabolism and CBF was preserved. A more complex but similar explanation can be proposed for the changes in PI. There are reports of a linear relationship between the PI and ICP [10,11] but from our data we could not distinguish whether changes in PI correlated more with T_core_ or ICP.

### What was already known

There is little information about how temperature affects cerebral hemodynamics in the clinical setting. Published data mainly focus on hypothermia and the most widely investigated setting is cardiac surgery, where a reduction in CBF-V during cooling and a parallel increase when temperature returned to baseline has been observed [12,13]. In one study 19 patients with chronic hepatitis C virus infection were subjected to experimental therapy with extracorporeal whole-body hyperthermia at 41.8°C for 120 minutes under propofol anesthesia; there was a decrease in cerebral oxygen extraction and an increase in CBF-V [14]. These findings take the same direction as our results, but our brain-injured patients had spontaneous episodes of fever, not passive hyperthermia. Hyperthermia and fever follow different pathways, so studies on hyperthermia cannot be directly translated into the fever setting.

Findings in healthy volunteers seem to contrast with our findings. CBF-V rose when T_core_ was lowered by an external system in healthy volunteers; this might be explained by concomitant changes in several other parameters, like arterial carbon dioxide partial pressure, which is the most widely investigated factor that influences CBF-V [15]. Some authors [16] also investigated the effect of exercise-induced hyperthermia on CBF-V and their results too appear to contrast with ours: CBF-V fell as temperature rose. Prolonged exercise causes hyperventilation, hence hypocapnia, which can reduce CBF and consequently CBF-V. We do not know how the other parameters change.

### Clinical implications

Changes in temperature affect cerebral hemodynamics, and potentially ICP. CBF-V, which estimates CBF, increases when temperature rises. Cerebral blood volume (CBV) is likely to increase as well. In patients with reduced intracranial compliance, small changes in CBV can be very important in terms of the effect on ICP. As temperature affects CBF-V, it must be taken into consideration for the interpretation of TCCDS data, as well as arterial partial pressure of carbon dioxide (P_a_CO_2_) [17-22], sedatives/analgesics [23,24] and induced hypertension [25], which are already known to change CBF-V. Moreover, changes in the PI parallel temperature and ICP; if this is the case, it can hardly serve as a useful basis for non-invasively estimating ICP, as previously proposed [10,11].

### Limitations

Our study has some limitations. First of all TCCDS does not directly quantify CBF but only estimates it. Autoregulation changes, which are of obvious importance, could not be studied. In addition, our analysis involved a limited number of patients. Even if we tried to change nothing other than the temperature, in some cases there were small changes in certain parameters. We also could not measure T_brain_ but only T_core_, which generally underestimates T_brain_.

## Conclusions

Body temperature affects cerebral hemodynamics, as estimated with TCCDS. CBF-V changes, consistent both after re-warming and after defervescence, may be interpreted as physiological CBF adaptations with potential impact on ICP. TCCDS offers an important insight into cerebral physiology, and is better understood when evaluated together with other measurements, such as temperature, ICP, CPP and SjO_2_.

## Key messages

- Body temperature affects cerebral hemodynamics
- Fever, a recognized entity that worsens brain injury, increases cerebral blood flow velocity and ICP
- Hypothermia decreases cerebral blood flow velocity
- We have to consider the effects of body temperature on cerebral blood flow velocity and pulsatility index when we use TCCDS as a diagnostic tool.

## Abbreviations

CBF: cerebral blood flow
CBF-V: cerebral blood flow velocity
CPP: cerebral perfusion pressure
ICP: intracranial pressure
MAP: mean arterial pressure
MCA: middle cerebral artery
NICU: neurosurgical intensive care unit
PI: pulsatility index
S_j_O_2_: oxygen saturation in the jugular vein
T_brain_: brain temperature
TCCDS: transcranial color-coded duplex sonography
T_core_: core body temperature
